# Radio Wave Propagation and WSN Deployment in Complex Utility Tunnel Environments †

**DOI:** 10.3390/s20236710

**Published:** 2020-11-24

**Authors:** Mikel Celaya-Echarri, Leyre Azpilicueta, Peio Lopez-Iturri, Imanol Picallo, Erik Aguirre, Jose Javier Astrain, Jesús Villadangos, Francisco Falcone

**Affiliations:** 1 School of Engineering and Sciences, Tecnologico de Monterrey, 64849 Monterrey, NL, Mexico; mikelcelaya@tec.mx; 2 Electric, Electronic and Communication Engineering Department, Public University of Navarre, 31006 Pamplona, Navarra, Spain; peio.lopez@unavarra.es (P.L.-I.); imanol.picallo@unavarra.es (I.P.); erik.aguirre@unavarra.es (E.A.); francisco.falcone@unavarra.es (F.F.); 3 Institute of Smart Cities, Public University of Navarre, 31006 Pamplona, Navarra, Spain; 4 Mathematical Engineering and Computer Science Department, Public University of Navarre, 31006 Pamplona, Navarra, Spain; josej.astrain@unavarra.es (J.J.A.); jesusv@unavarra.es (J.V.)

**Keywords:** wireless sensor networks, LoRaWAN, ZigBee, 3D Ray-Launching, tunnel environment, smart cities

## Abstract

The significant growth of wireless communications systems in the last years has led to the adoption of a wide range of applications not only for the general public but, also, including utilities and administrative authorities. In this context, the notable expansion of new services for smart cities requires, in some specific cases, the construction of underground tunnels in order to enable the maintenance and operation works of utilities, as well as to reduce the visual impact within the city center. One of the main challenges is that, inherently, underground service tunnels lack coverage from exterior wireless communication systems, which can be potentially dangerous for maintenance personnel working within the tunnels. Accordingly, wireless coverage should be deployed within the underground installation in order to guarantee real-time connectivity for safety maintenance, remote surveillance or monitoring operations. In this work, wireless channel characterization for complex urban tunnel environments was analyzed based on the assessment of LoRaWAN and ZigBee technologies operating at 868 MHz. For that purpose, a real urban utility tunnel was modeled and simulated by means of an in-house three-dimensional ray-launching (3D-RL) code. The utility tunnel scenario is a complex and singular environment in terms of radio wave propagation due to the limited dimensions and metallic elements within it, such as service trays, user pathways or handrails, which were considered in the simulations. The simulated 3D-RL algorithm was calibrated and verified with experimental measurements, after which, the simulation and measurement results showed good agreement. Besides, a complete wireless sensor network (WSN) deployment within the tunnels was presented, providing remote cloud data access applications and services, allowing infrastructure security and safety work conditions. The obtained results provided an adequate radio planning approach for the deployment of wireless systems in complex urban utility scenarios, with optimal coverage and enhanced quality of service.

## 1. Introduction

The evolution of wireless communication has led to their adoption in a wide range of applications, including utilities and administrative authorities. In addition, the growth in population, as well as in the services offered in cities, has led, in some cases, to the construction of underground tunnels in order to reduce the visual impact within the city center, as well as facilitating maintenance and operation works of utilities. In recent years, the underground sector has seen the use of wireless networking for certain projects, i.e., tunneling construction [1], but they do not make full use of the benefits offered by wireless communications. The utilization of wireless networking in the underground environment offers the potential for major benefits in productivity and safety. Currently, most of the communications infrastructure in operational tunnels such as transport tunnels are provided by cables. However, if it is an infrequently accessed tunnel, a wired communication infrastructure will not generally be available; therefore, the maintenance, upgrade and inspection of those tunnels will rely on wireless communications [2]. Nevertheless, one of the issues that local authorities have considered is the fact that, inherently, underground service tunnels lack coverage from exterior wireless systems, such as mobile networks or municipal wireless local area networks (WLAN), which can lead to potentially dangerous situations for maintenance personnel working within the tunnel. Therefore, wireless coverage should be deployed within the underground installation in order to guarantee real-time connectivity.

In emergency situations, such as fires, explosions or earthquakes, the normal wired communication infrastructure of an underground environment could be inoperable. Due to that, sometimes, it is necessary for the emergency services to have a wireless communication infrastructure following a serious incident. Rescue services normally are equipped with very high-frequency (VHF) or ultra-high-frequency (UHF) radio equipment, which is effective aboveground, but such radios have a very poor range in tunnels. The use of leaky feeder cables [3] is usually used to extend the coverage of VHF and UHF systems. However, the use of microwave frequencies provides a much greater range, without expensive leaky feeder systems, in such environments, and, also, they offer greater bandwidths. In addition, leaky feeders are also susceptible to damage during a fall or explosion. In [4], a system including both wired and wireless communications was described that enabled voice communication, as well as the exchange of photographs, video streams or other data, inside the tunnel. In [5], a wireless-based personnel tracking system to track people in an underground environment was presented, showing that the system can reduce the time taken for rescuers to reach any casualties.

The characterization of wireless channel behavior within complex heterogenous underground environments, such as utility tunnel systems, enables to perform link-balance estimations, which lead to coverage/capacity estimations. In this sense, an adequate estimation of losses is relevant in order to characterize the complex operation conditions, such as those given by high-obstacle density, with a high percentage of non-line-of-sight conditions and multipath propagation buildup owing to scatterers distributions within the scenarios under test or the effect of diffuse scattering, among others. This information is relevant in order to perform radio-planning tasks, which are required in order to design the network topology, node configuration and device-related parameters, such as antenna location/type. A radio-planning analysis was studied for different types of wireless networks (e.g., broadcasting systems, trunk radios, public land mobile systems, microwave transport links, etc.), considering aspects such as multi-variable optimization (energy consumption, node density and transmission rate as a function of the coverage area under analysis) and the impact of elements such as user density variation, estimation of cell edge conditions or interference background levels [6,7,8,9,10,11].

With the progressive adoption of wireless sensor networks, radio-planning tasks have been adapted to optimize network configuration [12,13,14,15,16,17,18]. In this case, the specific considerations given by the wireless sensor network (WSN) operation have to be taken into account, such as the random node location, the existence of predominant non-line-of-sight operation conditions or the inherent energy consumption and processing constraints, among others. In this way, a wireless channel analysis can be effectively employed in order to assess elements such as cooperative MAC schemes, optimized routing protocols or specific data aggregator policies in order to optimize the overall system operation. One of the main challenges in this case is to consider accurate radio channel conditions, given the large variability of node location and configuration within the wireless network layout, usually leading to the use of empirical/analytical-based channel estimation models. Radio propagation has been studied for different wireless systems within the interiors of galleries and tunnels [19,20,21,22], as well as for different tunnel geometries [23]. Other approaches have developed diffraction-based models, such as the alternate direction implicit parabolic equation method [24], boundary element method [25], numerical calculation of a vector parabolic equation [26] or application of the perturbation theory, among others. Another approach is based on the use of deterministic methods, such as ray launching (RL) or ray tracing (RT). One of the main issues of these approaches is the inherent divergence within the RL calculation as the distance increases between the transmit and receive antenna. In this case, solutions in scaled frequency models have been given by reconstructing the initial wave front of the source, achieved by ray density normalization [27]. One of the approaches adopted to perform wireless characterization within tunnels has been the adoption of dielectric waveguide models, in which the tunnel is emulated by a waveguide with dielectric walls [28]. These models, however, do not take into account the additional complexities, which appear in underground service tunnels, such as ducts, walkways or distribution trays, among others. Complete modeling can be achieved by employing full-wave electromagnetic simulation techniques, such as the finite difference time domain (FDTD) or frequency domain finite element techniques [29,30]. However, the computational cost of these techniques can be very high, due to the large size of the tunnel structures as compared to the wavelengths considered in conventional wireless communication systems. Moreover, the tunnels analyzed are usually devoted to road traffic or trains, with inherently larger cross-sections and lower structural complexity as compared with utility tunnels, with dense obstacle distributions.

In this paper, an in-house developed three-dimensional ray-launching (3D-RL) code is employed to analyze the wireless propagation in a real urban utility tunnel scenario at 868-MHz operation frequency. The aim of the article is a thorough analysis of the real results of measurements in the tunnels in order to obtain the optimal parameters for the simulation’s 3D-RL algorithm, so that, after their application, the simulated and experimental results show good agreement. By adequately modeling the tunnel gallery structure, a precise characterization for the complete extension of the tunnel system can be obtained, which can aid in the deployment of wireless communication and monitoring systems. Furthermore, a complete radio wave propagation analysis is performed, and a WSN deployment is proposed for the considered real underground utility tunnel system gallery. Finally, an infrastructure security and a work safety monitoring application prototype is developed in order to warrant work safety legislation.

**Figure 1:**
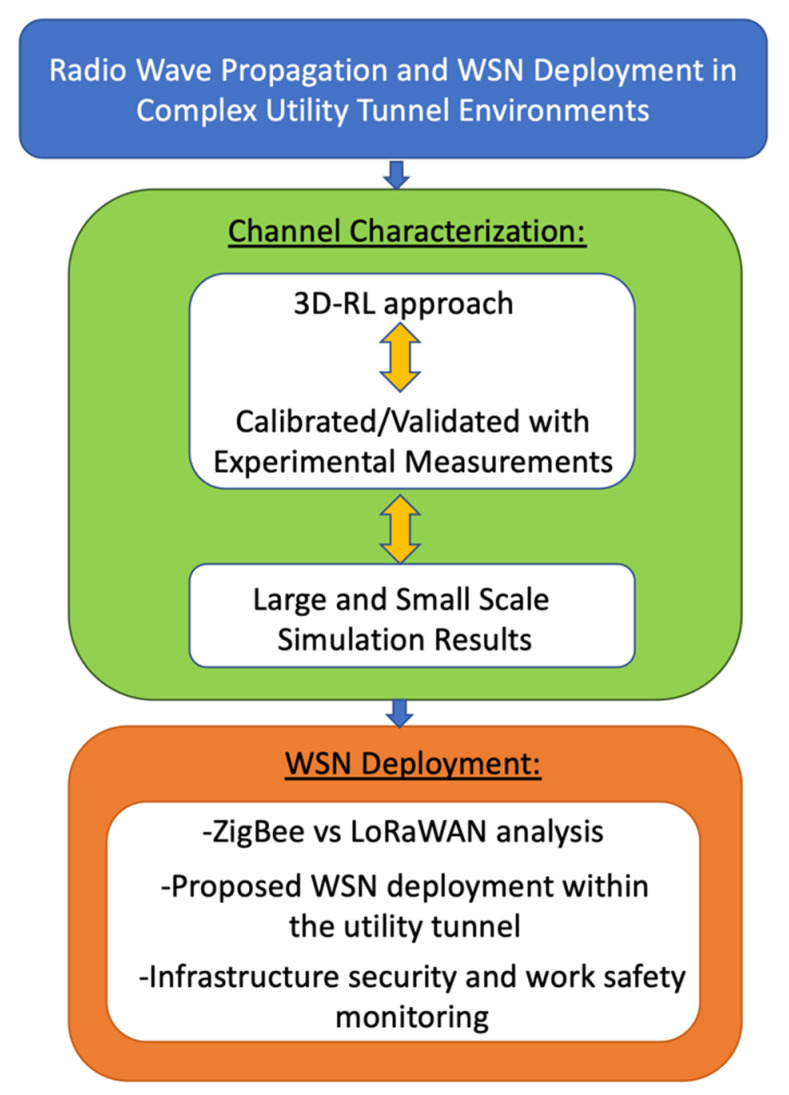
Schematic view of the structure of this work. WSN: wireless sensor network and 3D-RL: three-dimensional ray-launching.

The paper is organized as follows: the 3D-RL deterministic employed technique, as well as the considered real and simulated underground scenario are described in Section 2. Section 3 presents the radio channel analysis and the required convergence analysis due to the particular characteristics of the scenario. This analysis was performed compared with experimental measurements in the real utility tunnel in order to obtain the optimal simulation parameters. Once the optimal parameters were obtained, large- and small-scale simulation results within the tunnel were presented in order to characterize the radio wave propagation within this particular environment. In Section 4, the WSN deployment issues are discussed, and the infrastructure security and work safety monitoring application prototype are presented. Finally, the conclusions are given in Section 5. A schematic view of the structure of this paper is presented in Figure 1.

## 2. Materials and Methods

In this section, the in-house deterministic technique based on the 3D-RL approach is described, as well as the considered real and simulated underground utility tunnel scenario used for this analysis.

### 2.1. 3D Ray-Launching Technique

The in-house implemented RL algorithm, based on MATLAB programming environment, was used to analyze radio wave propagation in the considered underground tunnel scenario. The main difference between RL and classical RT methods is that, in the RL technique, the principle is that rays are launched in a solid angle and the rays arriving at the receiver are considered the true path. However, in classical RT techniques, the image of the transmitter or of the receiver is calculated, to be used to find the reflected and refracted paths.

**Figure 2:**
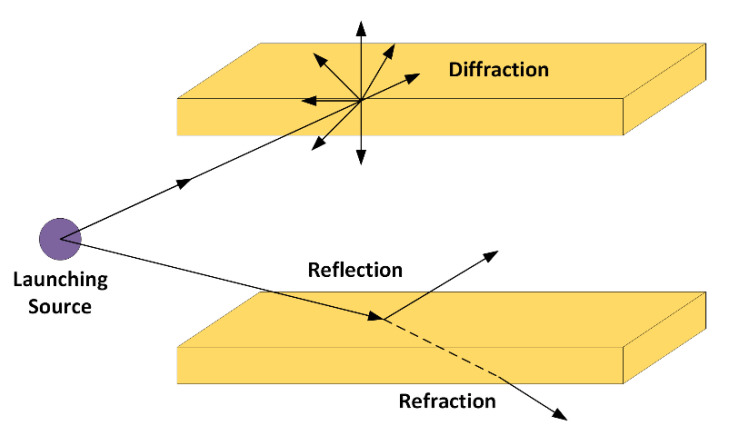
Principle of operation of the in-house 3D-RL algorithm.

The RL algorithm is based on geometrical optics (GO) and the geometrical theory of diffraction (GTD). To avoid the nonfield regions predicted by GO in the shadow areas, the diffracted rays are considered with the GTD and its uniform extension, called the uniform GTD (UTD). These diffracted rays are introduced to avoid the abrupt field changes and consider the proper field corrections. Electromagnetic phenomena such as reflection, refraction and diffraction are considered. The principle of the algorithm is that hundreds or thousands of rays are launched for a fixed position of the transmitter, which is an input parameter of the algorithm. The radiation pattern of the transmitter and receiver antennas can be introduced, with the aid of the elevation angle θ and the azimuth angle φ, considering the spherical coordinate system. Each ray has a different path in the considered scenario, and all the parameters related with its path are stored in a matrix. All the rays that achieve the receiver are considered in the calculation of the electromagnetic field at that point. The whole scenario is divided into a three-dimensional array of cuboids, with a different spatial resolution in the *x*-axis, *y*-axis and *z*-axis, depending on the specific characteristics and dimensions of the considered scenario. The basis is that, when a ray impacts an obstacle or a wall in the scenario under analysis, reflected and refracted rays are created, following Snell’s law. When a ray hits an edge, a new family of diffracted rays are created, according to the UTD. The methodology is shown in Figure 2.

As input parameters of the algorithm, the frequency of operation, number of multipath reflections and spatial and angular resolution are considered. In the simulations, the material properties of all the obstacles within the scenario are also considered, taking into account the conductivity and relative permittivity at the frequency range of operation of the system under analysis. The in-house implemented 3D-RL algorithm has already been validated in the literature for different complex scenarios for frequencies up to 6 GHz. Some scenarios that have been considered have been typical office scenarios [31,32], sports environments [33], vehicular communications [34] or WSNs in smart cities [35]. The detailed description of the algorithm can be found in [36].

### 2.2. Scenario Description

**Figure 3:**
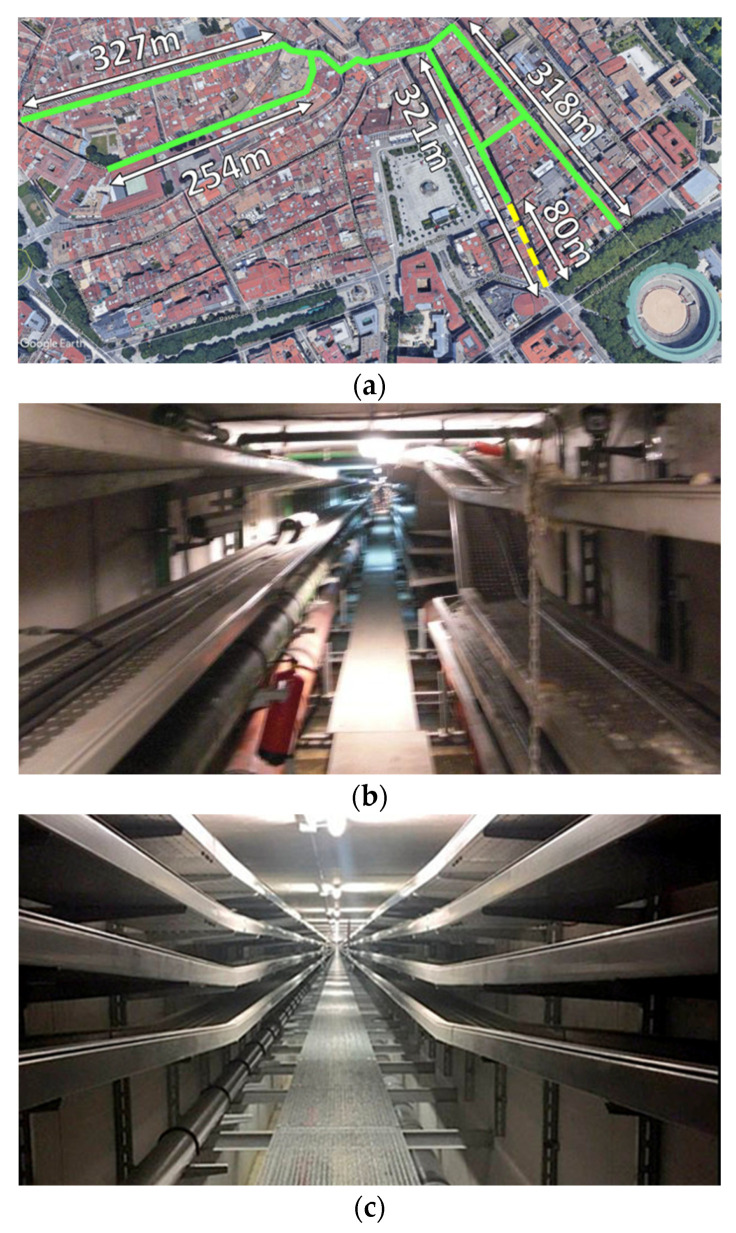
Underground utility tunnels under analysis. (**a**) Tunnel location on the map of the Old Town of Pamplona. (**b**) Picture of the tunnel where the measurements were taken. (**c**) Picture of a newer tunnel built in the neighborhood of Lezkairu in Pamplona.

In order to perform the radio wave propagation analysis within the tunnel, two scenarios were considered for the 3D-RL simulations: a real underground utility tunnel scenario located in the Old Town of Pamplona (Pamplona, Navarre, Spain) and a tunnel recreation of one of its gallery sections. The distribution of the considered experimental underground utility tunnels gallery is presented in Figure 3a with green lines on the map. As it can be seen from the figure, the tunnels have different sections, where the most remarkable characteristics are the different straight section lengths connected to one section with some intersections and slight curves. The dashed yellow line in the figure represents the straight tunnel section of 80-m-long where the experimental measurements were performed in order to calibrate the 3D-RL algorithm, as it is explained in Section 3. These tunnels were built in the early 2000s, under some specific streets, with the aim of easing maintenance operations. The tunnels consist of an outer layer of connected cement rings, with the inner part allocating several metallic trays for the corresponding utilities. The longest straight tunnel section is 327 m. A metallic hallway was also installed in order to provide access to the maintenance personnel, as can be seen in Figure 3b. Currently, they provide a route for the electrical connections, telephone connections, waste disposal and water distribution systems of the neighborhood. It is worth noting that slightly different utility tunnels have been built in newer neighborhoods of Pamplona, as can be seen in Figure 3c. Specifically, this picture corresponds to a recently repaired tunnel located in the suburb of Lezkairu, which began being built in 2008, and it has a total length of tunnels of 5600 m, plus 2300 m of sub-tunnels.

Regarding security and safety, the utility tunnels include common intrusion detection and antifire systems, with their corresponding local alarms. However, one of the main problems of these types of tunnels is the lack of wireless coverage within the tunnel, which initially was planned to be provided by external infrastructures, such as municipal Wi-Fi or mobile networks (3G/4G). However, in reality, these technologies are unable to provide coverage to all tunnel sections, as signal attenuation can be very high in some sections due to the different tunnel depths. In this sense, one of the main concerns of municipal authorities has been this lack of wireless coverage within the tunnels, as coverage is needed in order to provide security and safety to the maintenance staff. Thus, a specific indoor coverage system is required, and therefore, a complete radio planning analysis before the deployment of the wireless system is needed in order to secure coverage and provide good performance at an affordable cost.

**Figure 4:**
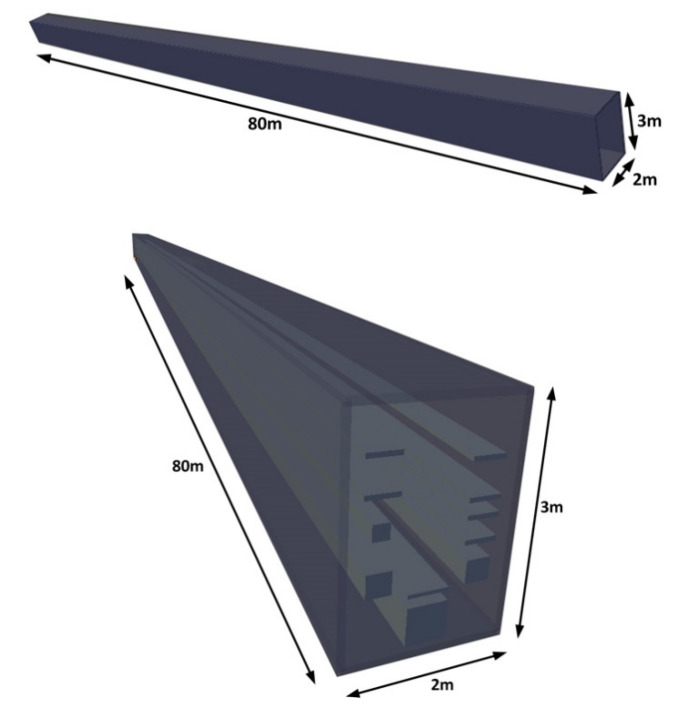
Schematic representation of the simulation scenario implemented to analyze the radio propagation losses within the underground utility tunnels.

As stated before, in order to characterize the variations of received power within the proposed utility tunnel, simulations by means of a 3D-RL code were performed. Material parameters were considered; basically, cement walls, aluminum trays and PVC tubes of different dimensions. A section of the simulation scenario is depicted in Figure 4, which corresponds with the same section where the experimental measurements were performed (see yellow dashed line in Figure 3a). The dimensions of the tunnel section are the following: width 2 m, height 3 m and length 80 m. Simulation results can be extended in length; however, due to measurement limitations, they will be considered up to the previously stated distance. The complete topological and morphological characteristics of this scenario were taken into account, prior visual inspection and measurement of dimensions within the tunnels. A schematic view of the employed simulation scenario is shown in Figure 4.

One of the particular aspects of this scenario is the fact that the tunnel is not a conventional tunnel (i.e., free hallway with walls that can be smooth or can present a certain degree of surface roughness and, hence, diffuse scattering), due to the fact that there is a set of service trays placed inside the tunnel. This leads to new interactions with the electromagnetic waves launched by the source, with strong multipath component generation given the large ratio between the length of the tunnel in comparison with the width of the tunnels, tray dimension and inter-tray distance. This condition implies the need for including more precise topological information of the scenario in order to consider the interaction, specifically for launched rays within a RL algorithm, such as the one employed and described in next subsection.

## 3. Radio Channel Analysis

### 3.1. 3D-RL Calibration and Validation

The underground tunnel scenario has distinctive characteristics that convert it in a particular scenario for radio wave propagation purposes. These peculiar characteristics are that it is an area that is considerably long (80-m-long, in this specific case) and extremely narrow (2-m width and 3-m height). It can be found in the literature [36,37], the convergence analysis of the in-house RL algorithm to obtain the optimal input parameters for the best performance of the algorithm in typical indoor complex scenarios. However, for this specific environment, those parameters are not the optimal, because the RL algorithm diverges due to the extremely narrow width of the scenario. The divergence of the algorithm means that there are a lot of cuboids of the scenario that do not have any ray if the same simulation parameters are used for this specific scenario. Due to that, the 3D-RL algorithm needs to be calibrated according to the experimental measurements to validate the results accuracy with the optimal input parameters. Therefore, a convergence analysis of the algorithm was done for this underground tunnel environment in order to characterize the 3D-RL convergence as a function of multiple parameters, such as cuboid dimensions, horizontal angular resolution (Δ*φ*), vertical angular resolution (Δ*θ*) or the maximum number of reflections. Due to the specific characteristics of the utility tunnel environment (i.e., strong reflections due to the partial conductor material presence and large obstacle density), the maximum number of reflections and the angular resolution of the launching rays was analyzed. For that purpose, the different number of multipath reflections and different number of launching rays were considered. Measurement results were compared with the different simulation results to find which simulation results achieved the best trade-off between precision and simulation computational cost, thus obtaining a calibrated 3D-RL technique that could be used for a radio-planning analysis in this type of complex underground environment. The considered frequency of operation was 868 MHz, emulating the operation of a typical LoRaWAN or ZigBee wireless communication system. The different cases for the convergence analysis, which was considered for the simulations of the underground tunnel scenario, are shown in Table 1.

**Figure 5:**
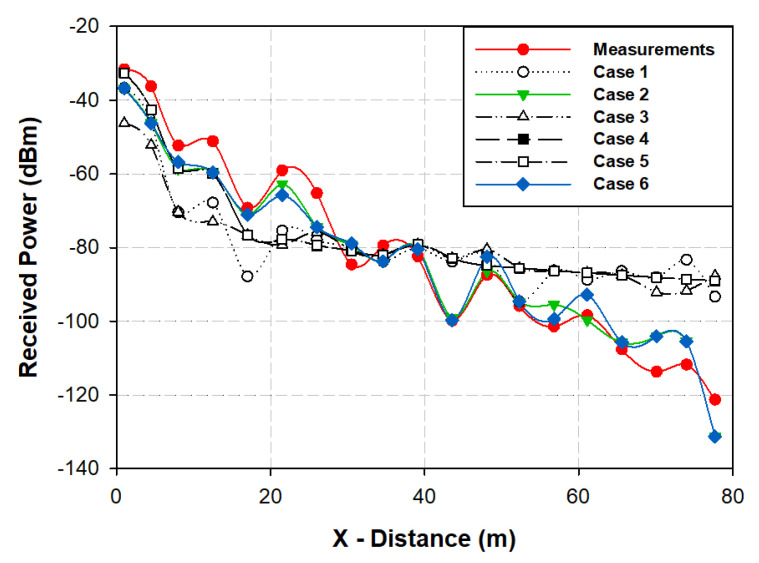
Comparison of different cases of simulation and measurement results for the service tunnel section under analysis.

Once the simulation results for the different cases were obtained, the validation and the corresponding calibration was obtained by performing radio channel measurements inside the tunnel section. The scenario in which the measurements were performed is a section of the utility underground tunnels in the city of Pamplona, which is depicted in Figure 3b. Measurements were carried out with the aid of an Agilent CSA N1996A to generate the radiofrequency (RF) signal and an Agilent FieldFox N9912A portable spectrum analyzer, both from Keysight Technologies. The transmitter was connected to a 5-dBi monopole antenna, whereas the receiver was connected to small vertical 0-dBi monopole antenna. The considered frequency of the operation was 868 MHz, with a transmitted power of −10 dBm. The transmitter antenna was placed at the beginning of the tunnel, located at the center with a height of 2 m, whereas the receiver points were placed at different distances in front of the transmitter along the tunnel. Measurements were performed with no users crossing the tunnels or other potential time-varying scatterers within the scenario. Results, compared with the different cases performed (shown in Table 1) in the 3D-RL simulations from the implemented scenario, are depicted in Figure 5. It can be seen from the picture that Cases 1, 3, 4 and 5 did not have good agreement with the measurements after 50-m distance approximately. This is because Cases 3, 4 and 5 considered larger angular resolutions than the other cases (one degree and 0.5 degrees of angular resolution) in comparison with 0.2 degrees in the other cases. In Case 1, the angular resolution was high, but the number of reflections was the lowest. It was shown that Case 1 did not have good agreement with the measurements. It was observed that Case 2 and Case 6 (both with angular resolutions of 0.2 degrees but different numbers of reflections considered) both had a good match with real measurements.

Table 2 shows the mean error and standard deviation of the comparison of the different simulation cases with the real measurements. It is also shown the simulation computational time of each one of the considered cases. It can be seen that Case 2 and Case 6 have the lowest mean error, as stated before. In Cases 1, 3, 4 and 5, the mean error increases considerably. With regards to the simulation computational time, it is observed that Case 6 has an increase of 186,861 s more than Case 2, with a slight decrease in the mean error. So, it can be concluded that, for the underground tunnel scenario, the optimal simulation parameters are Case 2, achieving a good trade-off between accuracy results and simulation computational burden.

### 3.2. Simulation Results

Once the calibration and verification of the 3D-RL algorithm for this singular scenario was achieved, the input optimal parameters, in terms of angular resolution and number of reflections, were applied in the simulations of the underground utility scenario. Large and small-scale radio wave propagation analyses were performed with the aid of simulations in order to provide a general signal propagation understanding within this complex and singular environment. The tunnel cross-sections have an important impact on the signal attenuation in this type of environment, as it is stated in [38]. In this sense, typical tunnel cross-sections can be classified in rectangular or circular shapes, where the tunnel width and height need to be considered when calculating the radio signal attenuation. Nevertheless, it has been shown in the literature [39,40] that cross-section dimensions within tunnels (i.e., different heights and widths of the tunnels) have a higher impact on the signal attenuation than distinct shapes, such as rectangular or circular. Therefore, the methodology, signal propagation analysis and WSN deployment presented in this work could potentially also be applied to cylinder tunnels, as long as tunnel cross-sections are of similar dimensions than the ones presented in this work. In fact, the cylindrical shape of the tunnel will generate slightly lower multipath propagation within the environment, as the number of reflections and diffractions within the surface of the tunnel will be lower.

**Figure 6:**
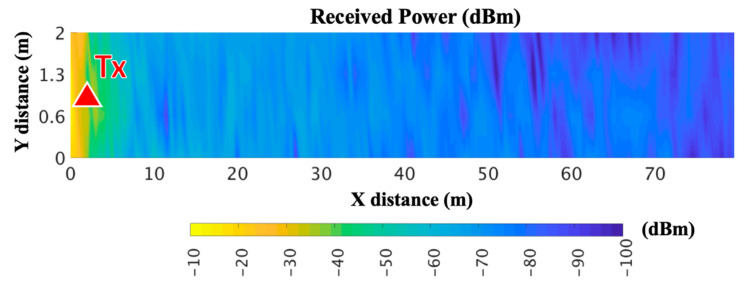
Radiofrequency (RF) power distribution for the XY bidimensional plane at 2-m height for 868 MHz.

Large scale propagation analysis was performed by means of the RF power distribution for the whole volume of the scenario. For that purpose, the transmitter antenna was placed at the beginning of the tunnel model, at a height of 2 m (represented by a red triangle in Figure 6). The same simulation parameters as in the experimental measurements were considered for the operational frequency and the transmitted power: 868 MHz and −10 dBm, respectively, emulating a ZigBee or LoRaWAN system at this specific frequency. Figure 6 shows the obtained RF power distribution estimations for the XY bidimensional plane at a height of 2 m (the same height as the transmitter antenna). It can be seen, the variability of received power along the distance, showing some spatial points with lower received power in the final part of the tunnel. The service trays on the sides of the tunnel generated a nonhomogeneous variation of the signal distribution levels along the tunnel distance.

**Figure 7:**
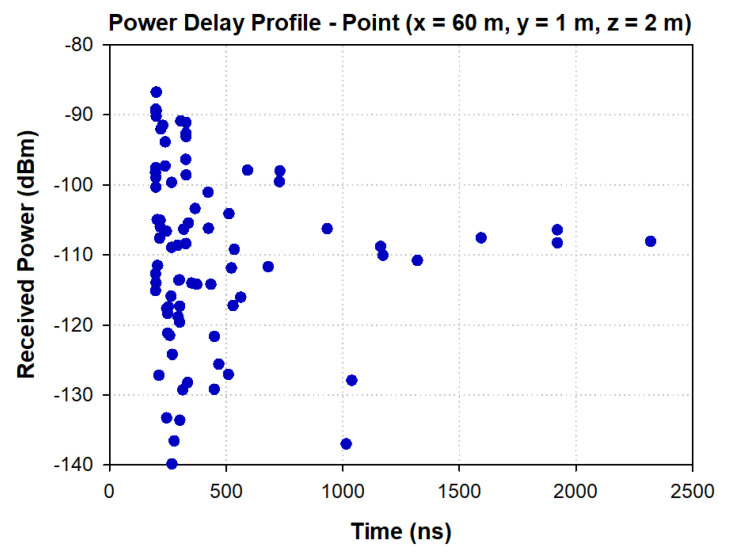
Power delay profile at a given cuboid located at the center (60 m, 1 m and 2 m) at 868-MHz frequency.

**Figure 8:**
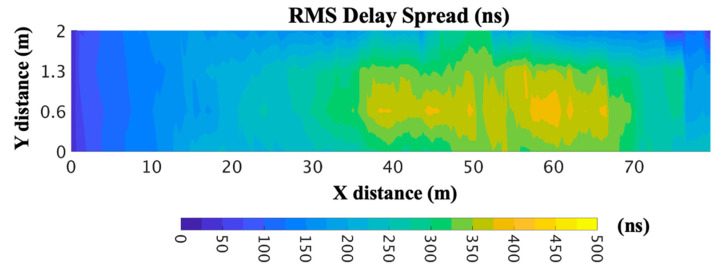
Root mean square delay spread (RMS DS) estimation within the utility tunnel for 2-m height for 868-MHz frequency.

To gain insight into the multipath behavior of the considered scenario, due to the rough surfaces of the walls and the service trays located in the tunnel, the power delay profile (PDP) is shown in Figure 7 for a specific point of the scenario, showing the high multipath trajectories within a time span of 200 and 2500 ns. The high multipath propagation within the environment can lead to signal fading in some spatial locations, which can lead to areas with a lack of coverage within the tunnel if the network design is not adequately performed. In addition, another parameter that can grossly quantify the multipath channel is the root mean square delay spread (RMS DS), referred also as $\sigma_{\tau }$, which shows the effects of dispersion and is determined from the PDP. The RMS DS for the considered utility tunnel environment is depicted in Figure 8. It is the square root of the second central moment of the PDP, defined as [41]:

(1) $$\sigma_{\tau }=\sqrt{\bar{\tau^{2}}-{\left(\bar{\tau }\right)}^{2}}$$

where

(2) $$\bar{\tau^{2}}=\frac{\sum_{k}a_{k\;}^{2}\tau_{k}^{2}}{\sum_{k}a_{k}^{2}}=\frac{\sum_{k}P\left(\tau_{k}\right)\tau_{k}^{2}}{\sum_{k}P\left(\tau_{k}\right)}$$

and

(3) $$\bar{\tau }=\frac{\sum_{k}a_{k}^{2}\;\tau_{k}}{\sum_{k}a_{k}^{2}}=\;\frac{\sum_{k}P\left(\;\tau_{k}\right)\tau_{k}}{\sum_{k}P\left(\tau_{k}\right)}$$

It must be pointed out that values for $\bar{\tau }$, $\bar{\tau^{2}}$ and $\sigma_{\tau }$ depend on the choice of the noise threshold to process the received power at each delay P(τ). The noise threshold used to depict the RMS DS shown in Figure 8 was −120 dBm, chosen to emulate the typical sensitivity of a ZigBee or LoRaWAN WSN that can be deployed in the tunnel environment.

It is observed that the RMS DS is higher in some areas closest to the center and the final part of the tunnel. This is because of the distribution of the service trays and tubes in the utility tunnel. Each spatial sample of the RMS DS is associated with a PDP. In this specific case, a multipath propagation contribution is higher in the central and final part of the tunnel, but it is necessary to consider all the spatial samples of the environment due to the influence of the topology and morphology of the specific scenario under consideration. Therefore, all the obstacles within the tunnel and the metallic structure of the service trays play an important role in the multipath propagation.

In order to have knowledge of the frequency domain channel description, the coherence bandwidth (CB) was obtained from the RMS DS for the considered scenario. The CB can be defined as the bandwidth over which the frequency correlation function is above 0.9; then, the CB is approximately $B_{c}\approx 1/50\sigma_{\tau }$; see [41].

**Figure 9:**
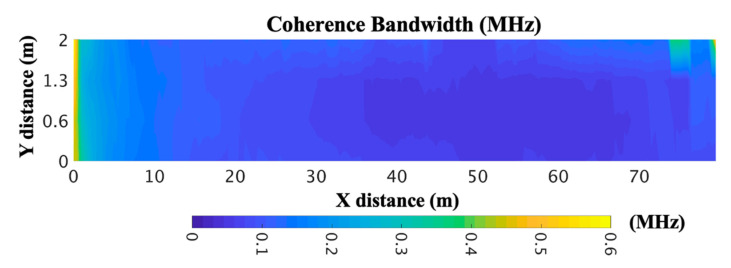
Coherence bandwidth within the considered utility tunnel for a 2-m height for 868-MHz frequency.

Figure 9 shows the CB for the considered utility tunnel showing values that vary from 0.01 to 0.6 MHz. It can be seen that the lowest values of RMS DS cause higher values of CB. Therefore, a receiver in those areas can encounter higher multipath interference.

## 4. WSN Deployment

In this section, a generic WSN deployment case for complex heterogeneous utility tunnels is presented considering environmental and propagation conditions by means of a calibrated 3D-RL simulation tool. A complete WSN deployment feasibility study is introduced focusing on the reliability of wireless communications technologies (Zigbee and LoRaWAN) within the considered scenario and, particularly, in terms of security management, assessing the human presence propagation impact over the system. Finally, an optimized WSN deployment is presented considering the morphology and topology of the real case study underground tunnel system environment and an infrastructure and work safety monitoring application prototype.

### 4.1. Safety Management and Occupational Risk Control

One of the main problems of working in tunnels and service galleries, which is aggravated by their length and narrowness, is the safety management and the occupational risks control. The health of the working environment of the maintenance staff must always be guaranteed. This includes monitoring temperature and humidity levels but, also, monitoring CO and CO_2_ levels; the presence of flammable or toxic gases and compounds and the presence of animals (rats, snakes, etc.), among others. In addition, mobility within the tunnel, the absence of obstacles or elements that hinder movement must be guaranteed. It should be borne in mind that employees, on many occasions, must move tools to carry out their maintenance, repair and installation tasks.

Current regulations related to labor risk prevention state that work must be done at least in pairs and desirable in work teams to minimize risks and prevent accidents. In addition, workers must notify the coordination center of the task to be performed; the starting time; the estimated duration; the working conditions (lighting, power supply cut, etc.); the possible risks and any other information that may help to ensure safety at work during the job performance. Hence, personnel monitoring implies that the staff will carry their own sensors (located in the inhalation zone and at head height) and must be able to interconnect to the monitoring network. Thus, the use of a wireless network is particularly advantageous in the latter case, as it offers a greater ease of work (hands-free) and greater mobility for workers. However, the presence of human beings within the tunnels can affect the radio propagation, even more considering their narrowness. Therefore, an adequate RF characterization and a complete risk control assessment must be performed in order to warrant compliance with occupational safety management within the utility tunnels.

### 4.2. Technology Analysis: ZigBee 868 MHz vs. LoRaWAN Range within the Tunnels

**Figure 10:**
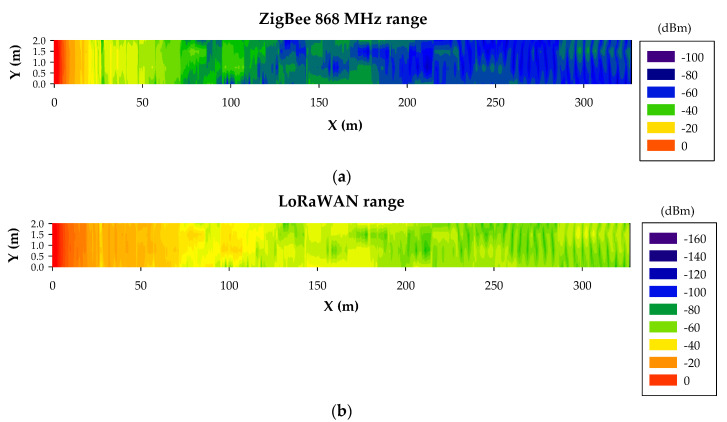
Bidimensional plane of the RF power level distribution at a height of 3 m for (**a**) ZigBee 868 MHz and (**b**) LoRaWAN.

3D-RL simulations were shown to be accurate for an optimized radio-planning analysis in this complex and particular underground environment. Based on these preliminary results, the coverage for both Zigbee and LoRaWAN wireless devices can be estimated (i.e., the number of wireless nodes required for the whole tunnel) considering their corresponding device sensitivities, which are around −100 dBm and −148 dBm, respectively. In order to provide clear insight, range estimations within the tunnel for both wireless technologies were obtained. For that purpose, a transmitter was placed on the ceiling at the beginning of the tunnel with a length equal to the longest tunnel section of the scenario under analysis (327 m, as it is presented in Figure 3a). The transmitted powers were set to 13 dBm and 14 dBm for ZigBee and LoRaWAN, respectively. As can be concluded from the results depicted in Figure 10, both wireless technologies covered the entire length of the tunnel, although LoRaWAN presented better results due to its better sensitivity (−148 dBm) compared to ZigBee (−100 dBm). Thus, in terms of wireless link distance within a straight tunnel of these characteristics, both wireless technologies could be employed.

Nevertheless, in the real environment, different phenomena can lead to channel impairments, such as the presence of people within the tunnels or worsening the radio propagation conditions. Therefore, a further study is required in order to provide safe, robust, reliable and efficient WSN deployment.

### 4.3. Scatterers Analysis: Human Presence within the Tunnel

An important factor that has to be assessed in terms of radio propagation in small cross-section utility tunnels is the presence of human beings. It is a well-known fact that human bodies create a shadowing effect [42], and in such a narrow environment like the underground tunnels presented in this work, this effect was expected to be especially significant. In order to have insight into this phenomenon, different simulations were performed using the calibrated 3D-RL simulation tool. Human body models are included in the 3D-RL software, emulating occupational personnel at the location of interest. This human body model was implemented for the simulation, considering the dimensions of a standard person and all the dispersive material properties of its different body parts (i.e., skin, bones, muscles, blood, different organs, etc.). A further description of the considered human body model, as well as a signal attenuation analysis for different parts of the human body, can be found in reference [43]. By including computational human body models within the considered scenario, the results of the human presence effect were obtained, allowing analysis and comparison. In the context of the utility tunnel, teams of up to three workers are generally sent into the tunnels for performing different security and maintenance tasks, in order to minimize the risks and prevent accidents due to municipal authorities’ requirements regarding occupational risk prevention legislation. Therefore, a complete analysis of the human presence within the tunnel was carried out considering different densities of human body models in different locations.

**Figure 11:**
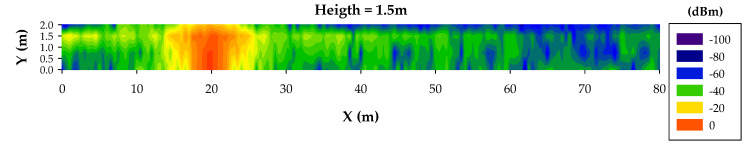
Bidimensional plane of the RF power level distribution at a 1.5-m height for the case without human presence.

First of all, the received power results of a specific scenario with no humans is presented in Figure 11, considering a transmitter located on the tunnel ceiling at *x*-axis = 20 m for reference and comparison purposes.

**Figure 12:**
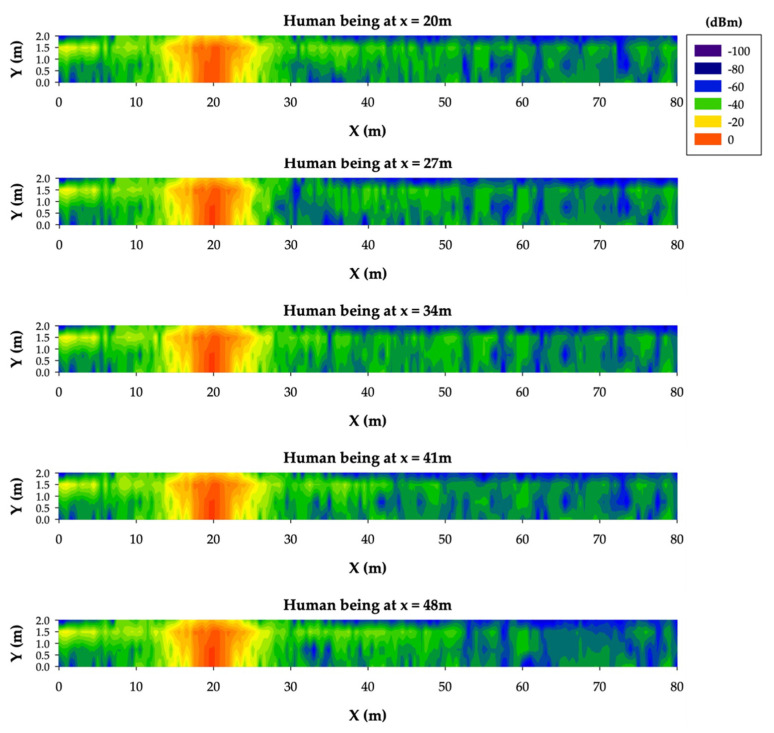
Bidimensional planes of the RF power level distribution at a 1.5-m height for different human being locations along the tunnel.

Then, a single human body model was included in the scenario at different *x*-axis locations in order to analyze the influence of the surrounding environment and the transmitter distance along the tunnel. The considered locations were the following: 20 m, 27 m, 34 m, 41 m and 48 m. In Figure 12, the corresponding RF power levels distribution results at 1.5-m height are presented. As expected, the major RF distribution changes are mainly in the area behind the location of the human body, due to the shielding effect.

**Figure 13:**
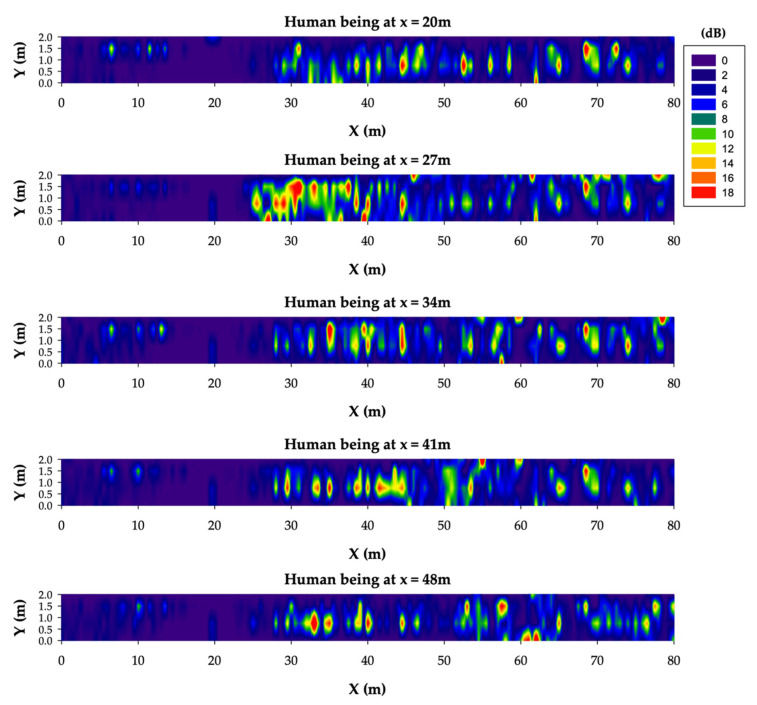
RF power level distribution differences between the case without a human presence and the cases with human beings at different locations along the tunnel.

In order to gain more insights into these results, Figure 13 shows the differences between the case without human presence (i.e., Figure 11) and the different case studies considering a single human body model at distinct locations (i.e., Figure 12). As can be clearly observed from Figure 13, the presence of a single worker within the tunnel in different locations leads to RF power level differences up to 18 dB. Although this difference is lower the higher the distance from the transmitter is, it has to be taken into account for an accurate range estimation. Thus, a margin of about 20 extra dBs should be added to the wireless communication range estimated in the previous subsection both for ZigBee and LoRaWAN technologies.

**Figure 14:**
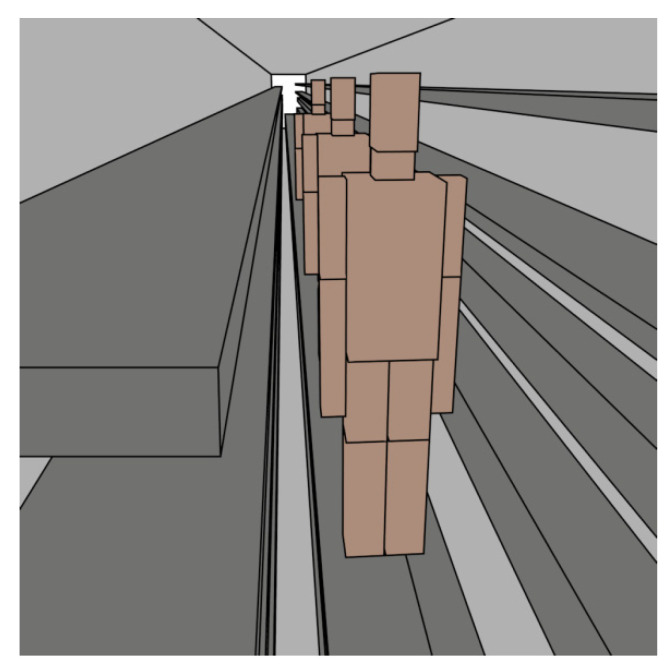
Inclusion of 3 computational human body models in the considered scenario for the 3D-RL simulation, emulating a maintenance team working in the utility tunnels.

Finally, extra simulations were carried out in order to assess if the obtained 20 dBs are enough when work teams of three people are within the utility tunnel. In Figure 14, a rendered view of the inclusion of the three human body models within the created tunnel scenario for the 3D-RL simulation is presented.

**Figure 15:**
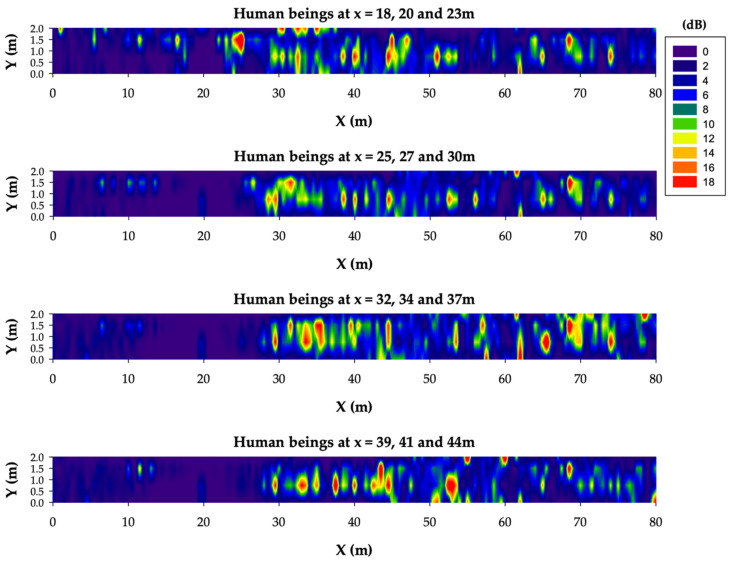
RF power level distribution difference between the case without people and the cases with a work team of 3 people at different locations along the tunnel.

Figure 15 presents the obtained estimation results in terms of the RF power level distribution differences between the case without a human presence and the case study with a three-worker team at different locations along the tunnel. From the results, a mild growth variation can be observed when comparing the three-worker team case study with the single-worker case study, and therefore, it can be stated that the previous estimated 20-dB margin is enough for a proper RF planification when considering teams of up to three workers within the tunnel.

**Figure 16:**
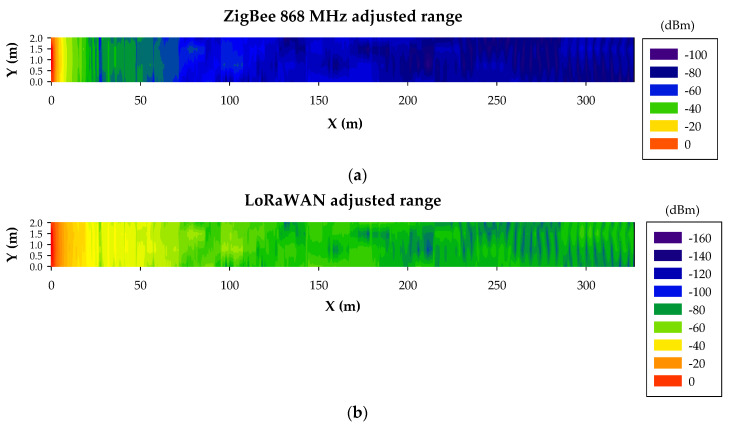
Bidimensional plane at a 3-m height representation of the RF power level distribution after applying the 20-dB margin due to the human presence for (**a**) ZigBee 868 MHz and (**b**) LoRaWAN.

In general, the obtained results regarding the study of the human presence within the tunnel show that a margin of about approximately 20 extra dBs is required in order to preserve the received RF power level above the sensitivity level, thus ensuring the wireless communication links between nodes, even when up to a three-worker team is considered within the tunnel. This extra margin corresponds to no need of extra range reduction estimated in the previous subsection analysis, as can be seen in Figure 16. However, since the sensitivity value of ZigBee is −100 dBm, in this case, a reduction of the distance between nodes is desirable in order to assure reliable wireless communication at every circumstance. This restriction was applied for the design of the WSN presented in the following subsection.

### 4.4. Proposed WSN Deployment

In this section, an optimized WSN deployment is proposed considering the morphology and topology of the real underground utility tunnel system. In general, the network topology gains a great importance for an adequate deployment and infrastructure design in this type of complex underground environments, since their particular morphology (for instance, tunnels in the presented case) could limit the use of the star topology (the typical one for LoRaWAN). From the previous results, it can be concluded that LoRaWAN technology presents a much higher range in terms of distance and better robustness with human presence due to its better sensitivity. Besides, it provides a high tolerance for interference, which is highly relevant in a confined scenario with the presence of tubes, grids and metal trays. However, considering the wireless system technologies’ capabilities and constraints, and the real utility tunnel system conditions in terms of connections, intersections and infrastructure location possibilities, there are several key factors that lead to the election of ZigBee technology to be deployed within the considered scenario. The first LoRaWAN limitation is the reduced transfer capacity (up to 242 bytes), but the most critical one is the network topology and its deployment in underground conditions. While the mesh topology provided by ZigBee gives the option of deploying one single gateway, the star topology of LoRaWAN leads to the deployment of several gateways due to the morphology of the tunnels. Furthermore, taking into account that the considered tunnels lack of communication systems infrastructure and coverage of external systems (such as the mobile network), the deployment of gateways presents serious difficulties, since they need external communication links in order to send the data of the underground tunnel network to the cloud. Therefore, the possibility of deploying a unique gateway is very advantageous.

**Figure 17:**
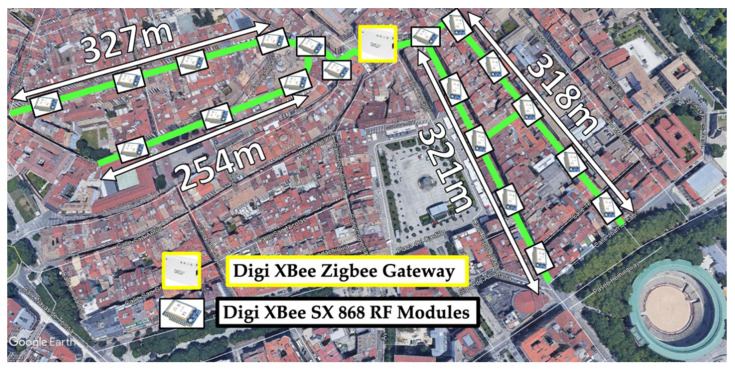
Schematic view of the proposed ZigBee 868-MHz WSN deployment.

For the proposed WSN deployment, a Digi International’s XBee Zigbee Gateway was selected, allowing GPRS communications. The gateway was designed to be installed in the center of the tunnel system for network performance optimization and located under a sewer, with the aim of facilitating the wireless communication with the mobile network. An efficient node of deployment was implemented with specific nodes located in key corners and intersections, considering the particular morphology of the tunnels and the maximum distance between the nodes obtained in the previous subsection analysis. Digi’s XBee SX 868 RF Modules were employed for the sensor nodes. A schematic view of the WSN deployment is depicted in Figure 17 representing the location of the ZigBee nodes and the gateway over the real utility tunnel system map.

### 4.5. Infrastructure and Work Safety Monitoring Application

In order to guarantee the security and protection of the infrastructure against unauthorized access, vandalism or sabotage, as well as to ensure occupational safety at work, a surveillance and monitoring system prototype was developed to allow infrastructure and work safety remote monitoring. In general, these types of systems usually include WSN, closed-circuit television (CCTV) and an antifire system, each of them with its own independent communication network. Although they could share the same communication network, for security and fault tolerance reasons, normally, three different communication networks are deployed, both wired and wireless. Adequate precautions, such as the use of shielded cables or fiber optics, should be incorporated when installing the complete wiring system to ensure the immunity to the effects of electromagnetic interference. However, sometimes, due to cost or deployment reasons, a wired network is not available, requiring a wireless communication network. In the analyzed real case, a fiber-optic network was available for the city’s security cameras, although no cameras were included inside the underground utility tunnel systems. If they were included, they could be connected to the fiber-optic network without further complications. Nevertheless, either special infrared cameras or visible spectrum cameras, in conjunction with presence sensors that activate the lighting system, should be included as well. This second option is more efficient in terms of infrastructure installation costs due to the difference in price of the cameras, but it has a higher probability of failure because it involves more elements (sensors and actuators). While, currently, no CCTV cameras are available in the galleries of the underground utility tunnel system, a wireless infrastructure and work safety monitoring application is proposed.

Regarding infrastructure security and work safety monitoring, WSN are devoted to monitoring possible leaks, zones or areas and personnel. When monitoring leaks (mainly water and gases), sensors should be installed as close as possible of the possible leakage in order to facilitate that the detection of the leak, if any, be as quick as possible. When monitoring zones/areas, detectors are regularly distributed throughout the area according to the potential risk (and its assessment) of leakage. On the one hand, some combustible gases, such as hydrogen or methane, are lighter than air, so the detector should be placed above the possible leak origin point or on the ceiling, where there is a high probability of accumulations of these gases. On the other hand, other gases such as propane or butane tend to move through the lower parts and can cover large areas. Therefore, in order to detect this type of gas in the fastest possible way, it is necessary to place the detectors in the lower parts of the installation, avoiding the contact with water. Finally, personnel monitoring is pivotal in order to provide work safety conditions, especially in underground environments. By means of a wireless network, surveillance and maintenance teamwork can be controlled and monitored along the galleries using wearables or worn body sensors, allowing free mobility and work service.

The development of an infrastructure-monitoring tool implies an adequate analysis and design of the whole system but, also, an adequate choice of the types of sensors, their locations, their configurations and their communication frequencies. In addition, the tool must allow the definition of alarm levels and the processes and procedures to be activated in case of alarm confirmation. Infrastructure monitoring will focus on both temperature and humidity control to prevent mold and bacteria buildup and to control potential sources of contamination. To do this, the humidity must be kept below 50% (desired values 30–50%). The same applies to dangerously high levels of carbon monoxide or dioxide in the air or nitrogen compounds (monoxide, dioxide and other nitrogen oxides) to prevent poisoning of the maintenance staff.

**Figure 18:**
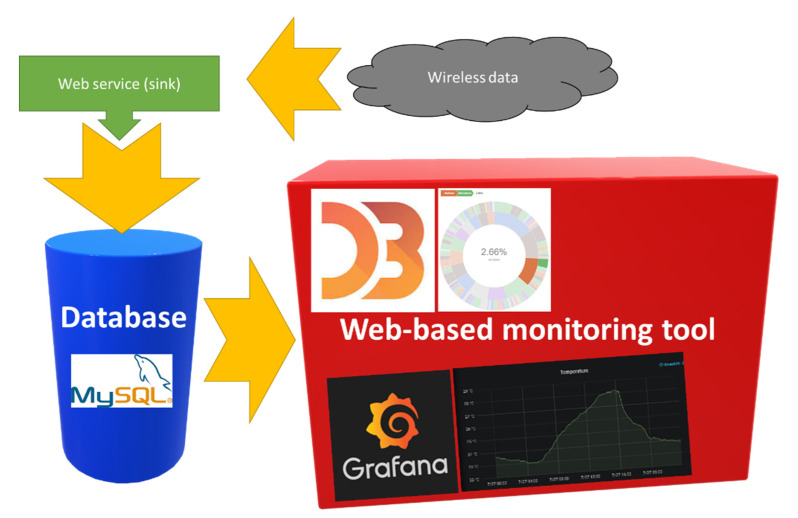
Architecture of the monitoring tool.

Considering the real case utility tunnel system and the proposed WSN deployment presented in the previous subsection, an infrastructure and work safety monitoring application prototype was developed in order to collect the data obtained by the sensors located in the gallery in real time and store them in a database allowing cloud computing. A schematic view of the monitoring tool architecture is depicted in Figure 18. The prototype was developed using open-source tools such as the Bootstrap framework [44] for the development of the web application Grafana [45] for the analytics and interactive visualization and the D3.js [46] JavaScript library for the production of dynamic and interactive data visualizations in web browsers. The system also includes a web service in charge of data collection, developed in Java, and a relational database (MySQL) [47].

**Figure 19:**
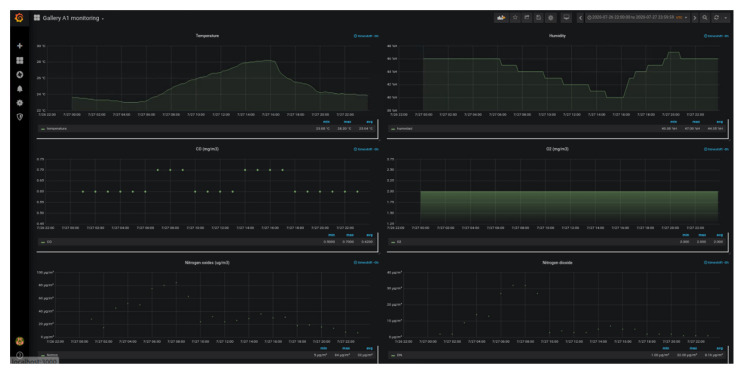
Time series monitoring by means of Grafana (iFrames).

**Figure 20:**
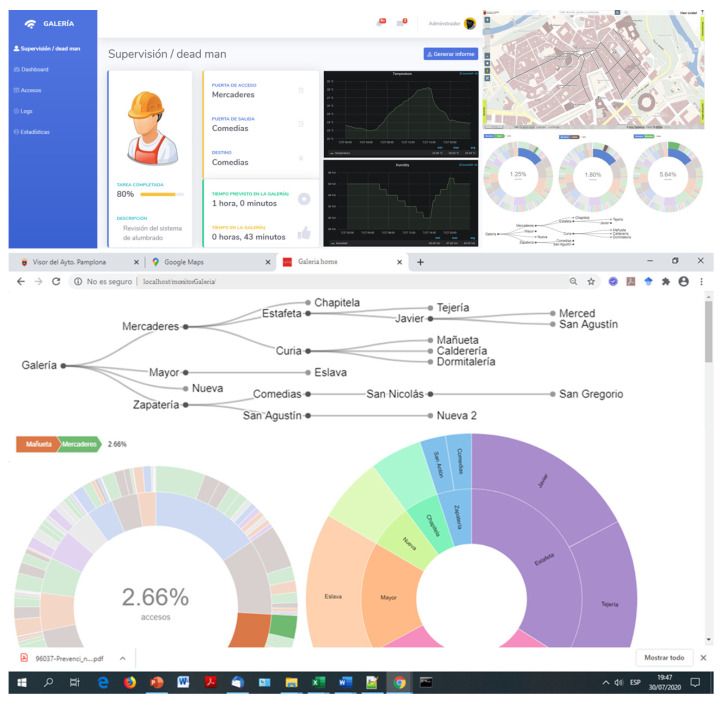
Web-based monitoring tool.

The design premises are security and use simplicity. The monitoring tool is responsive, which allows its use on mobile devices (smartphones and tablets), workstations or video walls. It embeds Grafana’s panels using iframes. On the one hand, there is a Grafana dashboard for the direct monitoring of the gallery’s health parameters, which allows the visualization of the data provided by the tunnel’s sensor network, which is depicted in Figure 19. On the other hand, the monitoring tool (see Figure 20), in addition to integrating the information provided by Grafana, includes the monitoring of the activities performed inside the gallery. These activities include dead man functionalities, monitoring of the task progress, access and exit points of the gallery, evacuation protocol and instructions in case of emergency, emergency acoustic and light signals activation, etc. The tool includes tree-based and sunburst visualization to describe the workflows.

The data collected by the WSN is stored, processed and analyzed in a private cloud. For that purpose, an Apache CloudStack was implemented over hypervisors VMware ESXI managed by a VMware vSphere. The resulting infrastructure allows the agile and easy deployment and management of the services. Besides, it allows computing orchestration, user and account management and provides data storage and data analytics. The proposed computing cloud is easily scalable and allows hot migrations according to the availability of the servers.

## 5. Conclusions

In this work, the radio wave propagation assessment and wireless system deployment for real urban utility tunnels was described. The impact of large obstacle density, given by the inclusion of elements such as service trays or handrails, as well as the presence of occupational staff within the underground utility tunnels, was analyzed by means of an in-house 3D-RL code. The calibration and validation of the 3D-RL algorithm for this singular scenario were presented by comparison with experimental measurements in the real scenario, showing good agreement between them. Once the optimal simulation parameters were obtained from the experimental measurements, the large and small-scale simulation results were presented to characterize the radio wave propagation within the tunnel. A complete volumetric analysis was performed in order to consider the location of wireless transceivers and enable them to be embedded within the elements of the tunnels. The system-level analysis considering the conventional transceiver parameters (ZigBee and LoRaWAN) was performed for an 868-MHz operational frequency, obtaining the initial deployment planning results. The use of higher frequencies could lead to a higher signal attenuation, and then, the coverage could be affected within the underground tunnel. Additionally, an infrastructure security and work safety monitoring application were developed in which the information was stored in a private Cloud. The proposed methodology enables to consider specific propagation losses in complex urban utility tunnels in order to deploy wireless communication systems with optimal coverage, minimum interference and an enhanced quality of service.

## Figures and Tables

**Table 1:** Different cases for the convergence analysis of the algorithm.

	Δ*φ* (rad)	Δ*θ* (rad)	Cuboids Size (m)	Number of Reflections	Number of Rays
Case 1	π/900	π/900	0.1	5	810,000
Case 2	π/900	π/900	0.1	7	810,000
Case 3	π/360	π/360	0.1	7	129,600
Case 4	π/180	π/180	0.1	7	32,400
Case 5	π/180	π/180	0.1	13	32,400
Case 6	π/900	π/900	0.1	13	810,000

**Table 2:** Comparison between different simulation cases.

	Case 1	Case 2	Case 3	Case 4	Case 5	Case 6
Mean Error (dB)	2.95	1.34	2.74	5.24	5.24	1.06
Standard Deviation (dB)	16.14	5.90	16.56	14.29	14.29	6.14
Computational Time (s)	52,664	78,459	4852	883	1656	265,320
